# The Ivy Street Hospital

**Published:** 1888-09

**Authors:** 


THE IVY STREET HOSPITAL, connected with the Southern Medical College, of Atlanta, we are informed, is being fitted up with new and improved facilities for the treatment of cases both in the medical and surgical line. A seperate Clinical Department will be established with an eye to the instruction of the medical class of the Southern Medical College, who will thus find increased advantages in attending this school.

